# Follistatin-Like 1 Attenuates Ischemia/Reperfusion Injury in Cardiomyocytes via Regulation of Autophagy

**DOI:** 10.1155/2019/9537382

**Published:** 2019-04-21

**Authors:** Weijun Yang, Qunjun Duan, Xian Zhu, Kaiyu Tao, Aiqiang Dong

**Affiliations:** Department of Cardiovascular Surgery, The Second Affiliated Hospital of Zhejiang University School of Medicine, No. 88 Jiefang Road, Hangzhou, China

## Abstract

**Background:**

The cardioprotective effect of FSTL1 has been extensively studied in recent years, but its role in myocardial ischemia/reperfusion injury (IRI) is unclear. In this study, we investigated the effect of FSTL1 pretreatment on myocardial IRI as well as the possible involvement of autophagic pathways in its effects.

**Methods:**

The effects of FSTL1 on the viability and apoptosis of rat cardiomyocytes were investigated after exposure of cardiomyocytes to hypoxia/ischemia by using the CCK-8 assay and Annexin V/PI staining. Further, western blot analysis was used to detect the effects of FSTL1 pretreatment on autophagy-associated proteins, and confocal microscopy was used to observe autophagic flux. To confirm the role of autophagy, the cells were treated with the autophagy promoter rapamycin or the autophagy inhibitor 3-methyladenine, and cell viability and apoptosis during IRI were observed. These effects were also observed after treatment with rapamycin or 3-methyladenine followed by FSTL1 administration and IRI.

**Results:**

FSTL1 pretreatment significantly increased viability and reduced apoptosis in cardiomyocytes exposed to hypoxia/ischemia conditions. Further, FSTL1 pretreatment affected the levels of the autophagy-related proteins and enhanced autophagic flux during IRI. In addition, cell viability was enhanced and apoptosis was decreased by rapamycin treatment, while these effects were reversed by 3-MA treatment. However, when the myocardial cells were pretreated with rapamycin or 3-methyladenine, there was no significant change in their viability or apoptosis with FSTL1 treatment during IRI.

**Conclusions:**

FSTL1 plays a protective role in myocardial IRI by regulating autophagy.

## 1. Introduction

The last decade is characterized by great improvements in living standards all over the world, but this trend is associated with an increase in the incidence of myocardial ischemia (MI), which has become a major cause of morbidity and mortality worldwide [1]. MI can cause arrhythmias, cardiac dysfunction, myocardial infarction, and even sudden death. Timely myocardial reperfusion is the most effective strategy for reducing acute myocardial ischemic injury and limiting the extent of MI, so as to protect patients from myocardial necrosis and other related complications after acute myocardial infarction [2]. Reperfusion strategies such as thrombolytic therapy and primary percutaneous coronary intervention have been developed in recent years, and they have significantly reduced mortality and infarct size and improved left ventricular function [3]. However, reperfusion itself can also lead to the destruction of cardiac structure or function, and this is generally referred to as myocardial ischemia/reperfusion injury (IRI) [4]. IRI is associated with myocardial cell apoptosis and necrosis and reduces the chances of cure after thrombolytic therapy [5]. Myocardial IRI also involves inflammation, oxidative stress, and calcium overload, among other factors [6]. However, there are currently no effective methods for treating cardiac IRI [7]. In order to reduce the risk of IRI, it is essential to develop new strategies and identify new targets for improving myocardial function.

Follistatin-like 1 (FSTL1), also referred to as TSC-36, is a member of the BM-40/SPARC/osteonectin family and encodes a secreted glycoprotein [8]. FSTL1 was originally identified in a murine osteoblastic cell line, where it was called transforming growth factor-*β*1 (TGF-*β*1)-induced protein [9]. In recent years, the significance of FSTL1 in the cardiovascular system has become increasingly clear. The concentration of circulating FSTL1 increases in cardiovascular conditions such as heart failure and severe coronary artery syndrome [10, 11]. FSTL1 has also been reported to inhibit myocardial hypertrophy caused by pressure overload and improve endothelial cell function and vascular remodeling in hypoxic-ischemic regions [12]. Moreover, experimental studies have shown that overexpression of FSTL1 alleviates myocardial injury in a mouse myocardial IRI model, and FSTL1 can reduce infarct size and myocardial cell apoptosis [13]. Similarly, in cultured neonatal rat cardiomyocytes, recombinant FSTL1 was found to reduce hypoxia/reoxygenation-induced apoptosis [14]. In contrast, deletion of FSTL1 from Tie2-cre mouse endothelial/endocardium resulted in mitral valve dysfunction, heart failure, and death [15]. Collectively, these data indicate that FSTL1 plays a clinically relevant role in the regulation of myocardial pathological processes and might be essential for the protection of the myocardium from IRI. It would be interesting to explore the pathways through which FSTL1 exerts these protective effects on cardiomyocytes.

Autophagy is an intracellular process that is responsible for the degradation of misfolded proteins or clearance of damaged organelles, so as to prevent potential cytotoxicity or intracellular stress and, in turn, prevent apoptosis [16]. Autophagy may be involved in the pathogenesis of a variety of human diseases [17], and, in the heart, autophagy occurs at basal levels under normal conditions, contributing to cellular homeostasis by cleaning up long-lived or excessive proteins and aged organelles. Thus, dysregulation of autophagy can have adverse effects on the myocardium [18, 19]. Autophagy has been shown to play an important role in the pathogenesis of IRI [18–20] and the regulation of IRI-induced myocardial cell death [20]. In addition, there is a lot of evidence to suggest that FSTL1 reduces myocardial cell apoptosis [13, 21, 22]. However, to date, there is little evidence linking autophagy with FSTL1 in the context of IRI. Therefore, the present study set out to investigate this possible link, and our findings did show that FSTL1 plays an important and protective role in IRI by regulating autophagy in cardiomyocytes.

## 2. Materials and Methods

### 2.1. Reagents

FSTL1 was obtained from Sino Biological (Beijing, China). The p62, beclin-1 LC3-I, and LC3-II proteins were from Abcam (Cambridge, MA, USA). The other antibodies used in this study were all from Cell Signaling Technology (Danvers, MA, USA). Rapamycin and 3-MA were purchased from Selleck (Shanghai, China).

### 2.2. Cell Culture

The rat cardiomyocyte cell line H9C2 was obtained from ATCC and was maintained in Dulbecco's modified Eagle medium (DMEM; Gibco Invitrogen, Carlsbad, CA, USA) containing 10% fetal bovine serum (Gibco) at 37°C in a humidified incubator with a 5% CO_2_ atmosphere.

### 2.3. In Vitro IRI Simulation

The H9C2 cells were maintained in serum-free DMEM for 2 h and treated with an ischemic buffer solution (118 mM NaCl, 24 mM NaHCO_3_, 1 mM NaH_2_PO_4_·H_2_O, 2.5 mM CaCl_2_·2H_2_O, 0.5 mM sodium EDTA·2H_2_O, 20 mM sodium lactate, and 16 mM KCl [pH 6.2]). After pregassing with 95% N_2_ and 5% CO_2_ for at least 5 min, the ischemic buffer solution was added to the cells. The cells were then placed in a sealed chamber containing a deoxygenation reagent; this resulted in the consumption of O_2_ and the production of CO_2_. Near-anaerobic conditions were produced with the AnaeroPack system (Mitsubishi Gas Chemical Co. Inc., Tokyo, Japan), which provided an O_2_ concentration of <1% and a CO_2_ concentration of ~5% within 1 h of incubation at 37°C. The cells were exposed to the near-aerobic conditions for 2 h, and then incubated under normal culture conditions (reperfusion) for 24 h [23].

### 2.4. Cell Viability Assay

H9C2 cells from the indicated control and experimental groups were plated on 96-well plates (3000 cells per well). Cell viability was assessed with the cell counting kit-8 (CCK-8, Beyotime Institute of Biotechnology). For the cell viability assay, 10 *μ*L of CCK8 was added to the cells, and their viability was measured at 450 nm with a microplate reader (SpectraMax 250; GE Healthcare Life Sciences, Pittsburgh, PA, USA). Three independent experiments were performed in quintuplicate.

### 2.5. Cell Proliferation Assay

H9C2 cells from the indicated control and experimental groups were assayed using the Click-iT 5-ethynyl-20-deoxyuridine (Edu) Imaging Kit (Invitrogen), in accordance with the manufacturer's instructions, and counterstained with Hoechst 33342. The percentage of proliferating cells in five random fields of view per slide was determined under an inverted fluorescence microscope (Olympus) and expressed relative to the percentage of proliferating cells in the untreated control group.

### 2.6. Flow Cytometry Analysis

Cell apoptosis was detected using the Annexin V/PI staining kit (BD Pharmingen) according to the manufacturer's instructions. The cells from the indicated control and experimental groups were washed twice with cold PBS and resuspended in 1× binding buffer at a concentration of 1 × 10^6^ cells/ml. Then, 100 *μ*l of the solution (1 × 10^5^ cells) was transferred to a 5-ml culture tube, to which 5 *μ*l of FITC Annexin V and 5 *μ*l of PI were added. The cells were incubated for 15 min at RT (25°C) in the dark, and 400 *μ*l of 1× binding buffer was added to each tube. Apoptosis was analyzed by flow cytometry within 1 h. Unstained cells, cells stained with FITC Annexin V (no PI), and cells stained with PI (no FITC Annexin V) were used for setting up the compensation and quadrants.

### 2.7. Western Blot Analysis

Cells from the indicated experimental and control groups were homogenized in lysis buffer (100 mM Tris-HCl [pH 8.0], 150 mM NaCl, 0.1% SDS and 1% Triton X-100) containing protease inhibitors on ice. Next, 30 *μ*g of each lysate was separated by SDS-PAGE and transferred to a PVDF membrane. After blocking with 5% nonfat milk, the PVDF membrane was exposed to the indicated primary antibodies at 4°C overnight. Then, the membrane was incubated with the indicated secondary antibodies for 2 h at room temperature and visualized using an enhanced chemiluminescence detection kit. A monoclonal antibody against *β*-actin was used as the loading control. The signals of the various bands formed on the membrane were analyzed using the Image J software (National Institute of Health, Bethesda, MD).

### 2.8. Fluorescence Microscopy Analysis

Cells from the experimental and control groups were transfected with an mRFP-GFP-LC3 adenovirus. After 48 h, the cells were fixed with 4% paraformaldehyde (Sigma, USA) and imaged under a laser confocal fluorescence microscope. The H9C2 cells were then examined for green (GFP) or red (mRFP) fluorescence. Autophagosomes were observed as yellow puncta and autolysosomes appeared as only red puncta in the merged images. Autophagic flux was determined based on the increase in the percentage of only red spots in the merged images.

### 2.9. ELISA

The supernatants were collected and stored at 4°C. Concentrations of CK-MB were detected using ELISA kits.

### 2.10. Statistical Analysis

Data are presented as mean ± standard of mean (SEM). All statistical analyses were performed using the SPSS (ver. 13.0) software. Comparisons between groups were analyzed with a two-tailed Student's T test or analysis of variance test (ANOVA). A P value of <0.05 was considered to indicate statistical significance.

## 3. Results

### 3.1. FSTL1 Pretreatment Inhibits Cell Apoptosis and Enhances Cell Viability in the H9C2 Cell Model of IRI

**Figure 1:**
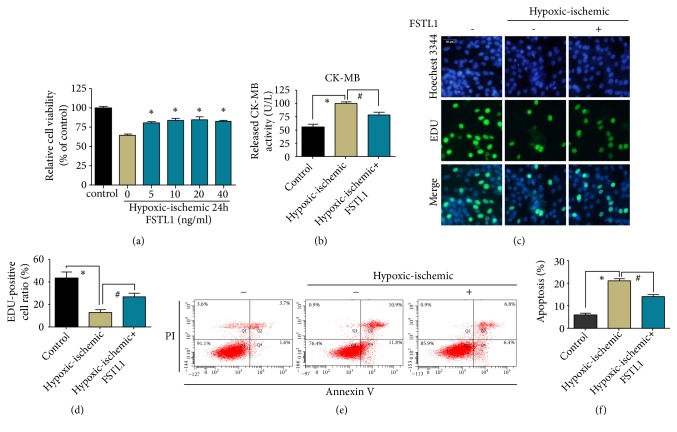
Effect of FSTL1 pretreatment on the viability and apoptosis of H9C2 cells exposed to hypoxia/ischemia. (a) H9C2 cells were pretreated with FSTL1 at concentrations of 0, 5, 10, 20, and 40 ng/ml, and cell viability was determined by the CCK-8 assay after exposure to hypoxia/ischemia conditions for 24 h. Cells cultured under normoxic conditions were used as the control. The concentration of FSTL1 used for the following pretreatments was 10 ng/ml. (b) The CK-MB levels were determined with a commercial ELISA kit through three independent experiments. *∗*P < 0.05 vs. the control group, #P < 0.05 vs. the hypoxia/ischemia group. (c) Representative immunofluorescence images of H9C2 cells pretreated or not pretreated with FSTL1 under hypoxia/ischemia conditions. Cells cultured under normoxic conditions were used as the control. Proliferating cells were stained with EdU, and the total cells were stained with Hoechst 3344. (d) The percentage of EdU-positive cells among the total cells was calculated and analyzed. *∗*P < 0.05 vs. the control group, #P < 0.05 vs. the hypoxia/ischemia group. (e) Apoptosis as assessed by flow cytometry after Annexin V/PI staining. (f) Percentage of apoptotic cells. *∗*P < 0.05 vs. the control group, #P < 0.05 vs. the hypoxia/ischemia group. All experiments were repeated at least three times.

To address the potential role of FSTL1 in myocardial IRI, we established a cellular IRI model by exposing H9C2 cells to hypoxia in serum-free and sugar-free medium and then reoxygenation in normal medium. The optimal concentration of FSTL1 was determined by pretreatment of the cells with different concentrations of FSTL1 for 24h and hypoxic/ischemic for 24 h. Cell viability under hypoxic/ischemic conditions was obviously lower than that under normal culture conditions. Cell viability was higher in the FSTL1-pretreatment groups than in the untreated group (Figure 1(a)). Further, cell viability in the 20 ng/ml FSTL1 group was not significantly higher than that in the 10 ng/ml FSTL1 group. Therefore, subsequent experiments were performed using FSTL1 at a concentration of 10 ng/ml (Figure 1(a)).

Creatine kinase MB (CK-MB) is a diagnostic marker of myocardial tissue injury [24]. In this study, the CK-MB content in the supernatant of the myocardium was determined by enzyme-linked immunosorbent assay (ELISA). The CK-MB level in the hypoxia/ischemia group was significantly higher than that in the control group. Further, the CK-MB level in the FSTL1-pretreated group was significantly lower than that in the hypoxia/ischemia group (Figure 1(b)). These findings indicate that FSTL1 had a protective effect on the cells during the process of IRI.

Next, we detected the effect of FSTL1 on apoptosis and proliferation in the IRI model cells. The proportion of EdU-positive cells in the hypoxia/ischemia group was significantly lower than that in the control group, but pretreatment with FSTL1 significantly enhanced cell proliferation (Figures 1(c) and 1(d)). Furthermore, Annexin V/PI staining for cell apoptosis (Figures 1(e) and 1(f)) showed that the apoptosis rate in the hypoxia/ischemia group was significantly higher than that in the control group. However, FSTL1 pretreatment resulted in a significant decrease in the apoptotic rate compared with the hypoxia/ischemia group. These data indicate that FSTL1 protects cardiomyocytes from undergoing apoptosis during IRI.

### 3.2. FSTL1 Pretreatment Promotes Autophagy in H9C2 Cells

**Figure 2:**
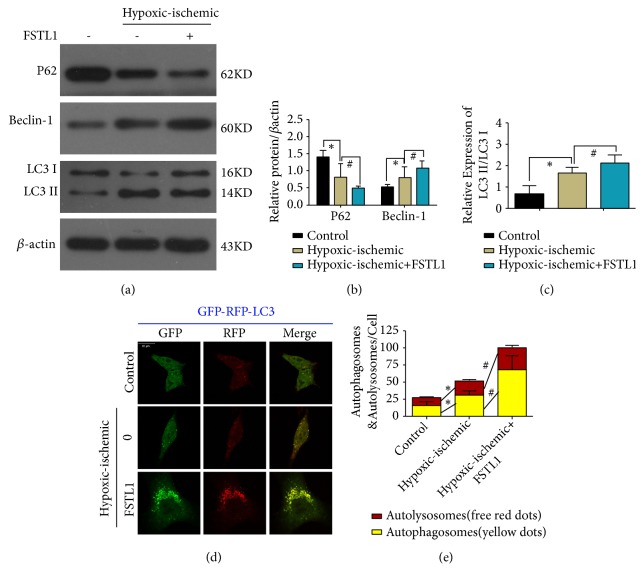
Effect of FSTL1 pretreatment on autophagy induction in H9C2 cells exposed to hypoxia/ischemia. (a) Western blot shows the effects of FSTL1 pretreatment on p62, beclin-1, and LC3 expression. (b) Quantitation of P62 and beclin-1 expression was performed using three independent experiments. *∗*P < 0.05 vs. the control group, #P < 0.05 vs. the hypoxia/ischemia group. (c) Quantitation of LC3 was performed using three independent experiments. *∗*P < 0.05 vs. the control group, #P < 0.05 vs. the hypoxia/ischemia group. (d) H9C2 cells under different conditions were cotransfected with RFP-LC3 and GFP-LC3, and viewed under a confocal microscope. The yellow dots represent autophagosomes. The red dots represent autolysosomes. (e) The level of autolysosomes and autophagosomes was analyzed. The red represents autolysosomes. The yellow represents autophagosomes. *∗*P < 0.05 vs. the control group, #P < 0.05 vs. the hypoxia/ischemia group. All experiments were repeated at least three times.

To explore the possible links between autophagy and FSTL1, we examined the effect of FSTL1 on autophagy-associated proteins in H9C2 cells exposed to hypoxia/ischemia conditions. Western blotting showed that the level of Beclin-1 protein and the LC3-II/I ratio was higher and the level of the P62 protein was lower in the hypoxia/ischemia group than in the control group. In contrast, in the FSTL1-pretreated group, the opposite findings were obtained in relation to the control group (Figures 2(a)–2(c)). Moreover, the level of LC3 immunofluorescence in the FSTL1 group was significantly higher than that in the hypoxia/ischemia group (Figures 2(d) and 2(e)). These results indicate that FSTL1 plays a proautophagic role in H9C2 cells.

### 3.3. Effect of Autophagy on the Viability and Proliferation of H9C2 Cells

**Figure 3:**
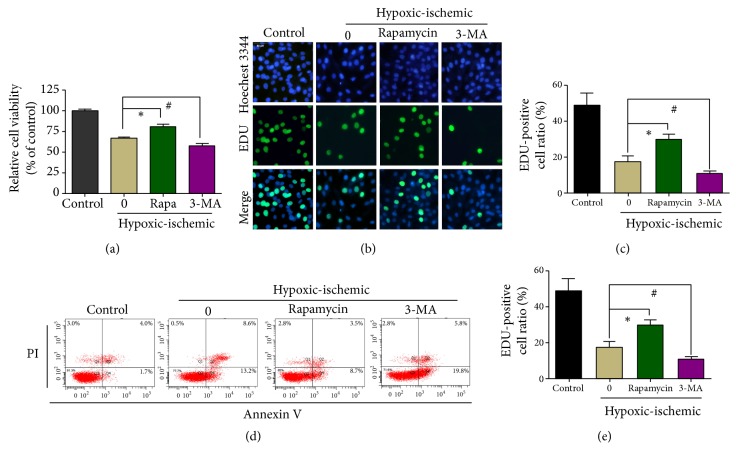
Effect of autophagy after FSTL1 pretreatment on the viability and apoptosis of H9C2 cells exposed to hypoxia/ischemia conditions. (a) Cell viability was determined with the CCK-8 assay under different conditions. *∗*P < 0.05, #P < 0.05 vs. the hypoxia/ischemia group. (b) Representative immunofluorescence images of H9C2 cells exposed to different conditions. Proliferating cells were stained with EdU, and the total cells were stained with Hoechst 3344. (c) The percentage of EdU-positive cells among the total cells was calculated and analyzed. The 0 group: the cells had no treatment under hypoxic/ischemic. *∗*P < 0.05, #P < 0.05 vs. the hypoxia/ischemia group. (d) Apoptosis as determined by flow cytometry after Annexin V/PI staining. The 0 group: the cells had no treatment under hypoxic/ischemic. (e) Percentage of apoptotic cells. The 0 group: the cells had no treatment under hypoxic/ischemic. *∗*P < 0.05, #P < 0.05 vs. the hypoxia/ischemia group. All experiments were repeated at least three times.

To investigate the effect of autophagy on H9C2 cells exposed to hypoxia/ischemia, the cells were treated with the autophagy activator rapamycin and the autophagy inhibitor 3-methyladenine (3-MA). The H9C2 cells were treated with either rapamycin or 3-MA for 24h before induction of hypoxia/ischemia. The concentration of 3-MA was 25 u M and the concentration of rapamycin was 100 nM (FiguresS1 and S2). The CCK-8 assay showed that exposure to hypoxia/ischemia led to a decrease in cell viability in comparison with the control cells (Figure 3(a)). However, treatment with rapamycin before induction of hypoxia/ischemia significantly enhanced cell viability compared with the untreated cells, while treatment with 3-MA had the opposite effect (Figure 3(a)). In agreement with the cell viability findings, the EdU incorporation assay showed that before the induction of hypoxia/ischemia, the proliferation rate of the rapamycin-treated cells was significantly higher than that of the control cells, while that of the 3-MA-treated cells was significantly lower than that of the control cells (Figures 3(b) and 3(c)). In contrast, before the induction of hypoxia/ischemia, the apoptosis rate of the rapamycin-treated cells was significantly lower while the apoptosis rate of the 3-MA-treated cells was significantly higher than that of the control cells (Figures 3(d) and 3(e)). Our data indicate that rapamycin could protect H9C2 cells during IRI (by promoting autophagy), whereas 3-MA aggravated cell injury.

### 3.4. Effect of FSTL1 on the Viability of H9C2 Cells after Pretreatment with Rapamycin or 3-MA

**Figure 4:**
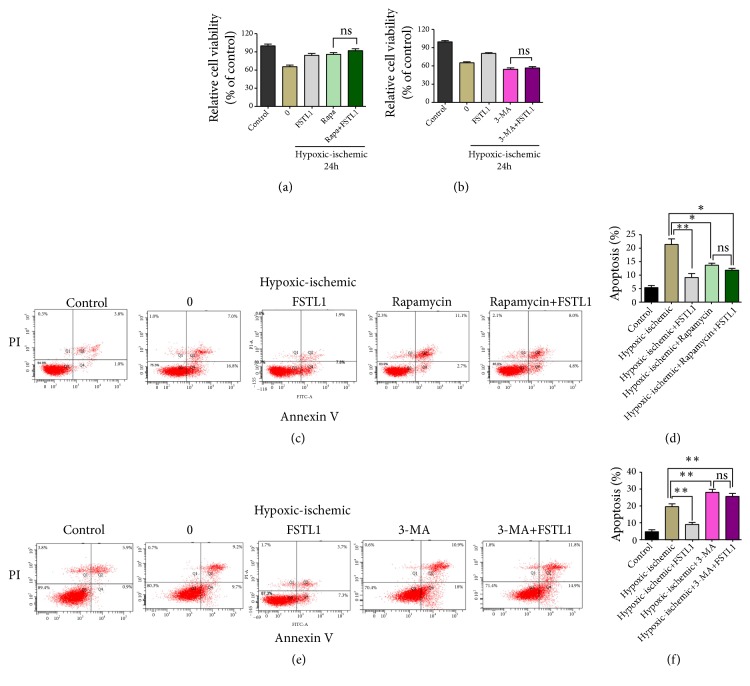
Effect of FSTL1 treatment after rapamycin or 3-MA pretreatment on the viability and apoptosis of H9C2 cells exposed to hypoxia/ischemia conditions. (a) and (b) Cell viability was determined with the CCK-8 assay under different conditions. The 0 group: the cells had no treatment under hypoxic/ischemic. (c) Apoptotic cells as determined by flow cytometry after Annexin V/PI staining. (d) Percentage of apoptotic cells. *∗*P < 0.05, *∗∗*P < 0.01 vs. the hypoxia/ischemia group. (e) Apoptotic cells as determined by flow cytometry after Annexin V/PI staining. (f) Percentage of apoptotic cells. *∗*P < 0.05, *∗∗*P < 0.01 vs. the hypoxia/ischemia group. All experiments were repeated at least three times.

To investigate the effect of rapamycin or 3-MA combined with FSTL1 during IRI, H9C2 cells were pretreated with rapamycin or 3-MA, treated with FSTL1, and then subjected to IRI. Surprisingly, under hypoxic/ischemic conditions, there was no significant difference in cell viability between the rapamycin and rapamycin+FSTL1 group (Figure 4(a)). Similarly, under hypoxia/ischemia conditions, there was no significant difference in cell viability between the 3-MA group and 3-MA+FSTL1 group (Figure 4(b)). Consistent with this finding, the apoptosis experiments showed that there was no difference in the apoptosis rate with or without FSTL1 pretreatment (Figures 4(c)–4(f)). These results imply that the cardioprotective effects of FSTL1 involve an autophagic component.

## 4. Discussion

In the present study, our findings show that FSTL1 can significantly enhance cell viability and decrease cell apoptosis of cardiomyocytes under hypoxia/ischemia conditions. The mechanistic experiments revealed that the cardioprotective effect of FSTL1 was mediated via its effects on autophagy. Taken together, the findings of our study indicate that FSTL1 pretreatment may be a promising new therapy for protection against heart IRI.

Early and successful myocardial reperfusion after an acute myocardial infarction is the most effective strategy for salvaging the myocardium and improving clinical outcomes. But reperfusion can cause additional cell death and increased infarct size. Follistatin-like 1 (FSTL1) is a secreted glycoprotein involved in a series of physiological and pathological processes. FSTL1 has been increasingly recognized as a potent cardiac protection factor [21, 25]. In this study, we established an ischemia/reperfusion injury model of cultured cells by inducing hypoxia in a serum- and glucose-free medium, followed by reoxygenation in normal culture medium. We observed that pretreatment with FSTL1 could significantly enhance cell viability and reduce cell apoptosis of H9C2 cells under hypoxia/ischemia conditions. These results demonstrate the cardioprotective role of FSTL1 in IRI. Therefore, we next explored the possible mechanisms of myocardial IRI that FSTL1 might be involved in.

Since many studies have suggested that autophagy can affect the pathogenesis of IRI [6, 26, 27], we decided to investigate whether FSTL1 exerts its cardioprotective effects in IRI via autophagic pathways. We used the LC3-II/I ratio, p62, and Beclin-1, which are widely used markers of autophagy. p62 levels inversely correlate with autophagy activity, while the LC3-II/I ratio and beclin-1 levels directly correlate with autophagic activity. We found that the beclin-1 protein level and LC3-II/I ratio increased while the P62 level decreased in the IRI group that was administered FSTL1, in comparison with the IRI group that was not administered FSTL1. Moreover, pretreatment with FSTL1 was also associated with an increase in LC3 immunofluorescence. These results indicate that autophagy induced by FSTL1 preconditioning has a protective role in the myocardium. Furthermore, we observed that pretreatment with rapamycin, an activator of autophagy, led to a significant increase in cell proliferation and reduction in cell apoptosis of H9C2 cells under hypoxic/ischemic conditions. This further confirms that autophagy has a protective effect on cardiomyocytes exposed to hypoxic/ischemic conditions.

Although autophagy is important for the maintenance of homeostasis, it can be a double-edged sword under certain conditions [28]. In the case of IRI, autophagy can prevent damaged mitochondria from releasing cytotoxic substances and thereby regulate the inflammatory processes and prevent further myocardial damage [29]. However, uncontrolled induction of autophagy in response to IRI may result in excessive cardiomyocyte apoptosis and aggravate the injury. In our study, to confirm the protective effect of autophagy activation in cardiomyocytes, we investigated the effects of promoting autophagy with rapamycin and inhibiting autophagy with 3-MA. Rapamycin treatment provided significant protection against IRI, as evidenced by the increase in cell viability and proliferation. By contrast, 3-MA treatment had the opposite effects. In addition, rapamycin treatment prior to FSTL1 administration did not further enhance FSTL1-mediated protection against IRI, while 3-MA treatment prior to FSTL1 administration did not aggravate heart injury. These data support our hypothesis that FSTL1 plays a protective role in IRI by regulating autophagy.

In conclusion, our findings demonstrate the crucial role of FSTL1 in protecting cardiomyocytes against myocardial IRI. Thus, pretreatment with FSTL1 may prove to be a new therapeutic strategy to protect the myocardium from IRI. We also found that FSTL1 exerted these effects via autophagic mechanisms, but we did not explore which autophagic pathways may be involved. This would be an interesting line of research for the future.

## 5. Conclusions

FSTL1 plays a protective role in myocardial IRI by regulating autophagy.

## Data Availability

All the data used to support the findings of this study are included within the article.

## Supplementary Materials

Supplementary Materials Figure S1: the effect of 3-MA on cell viability in H9C2 cell. Figure S2: the effect of Rapamycin on cell viability in H9C2 cell.
