# Supplementary material for: Follistatin-Like 1 Attenuates Ischemia/Reperfusion Injury in Cardiomyocytes via Regulation of Autophagy
Source: Biomed Res Int. 2019 Apr 21;2019:9537382. doi: 10.1155/2019/9537382 (PMC6500619; doi:10.1155/2019/9537382)
Supplement: Supplementary Materials — Figure S1: the effect of 3-MA on cell viability in H9C2 cell. Figure S2: the effect of Rapamycin on cell viability in H9C2 cell. [file 9537382.f1.docx]

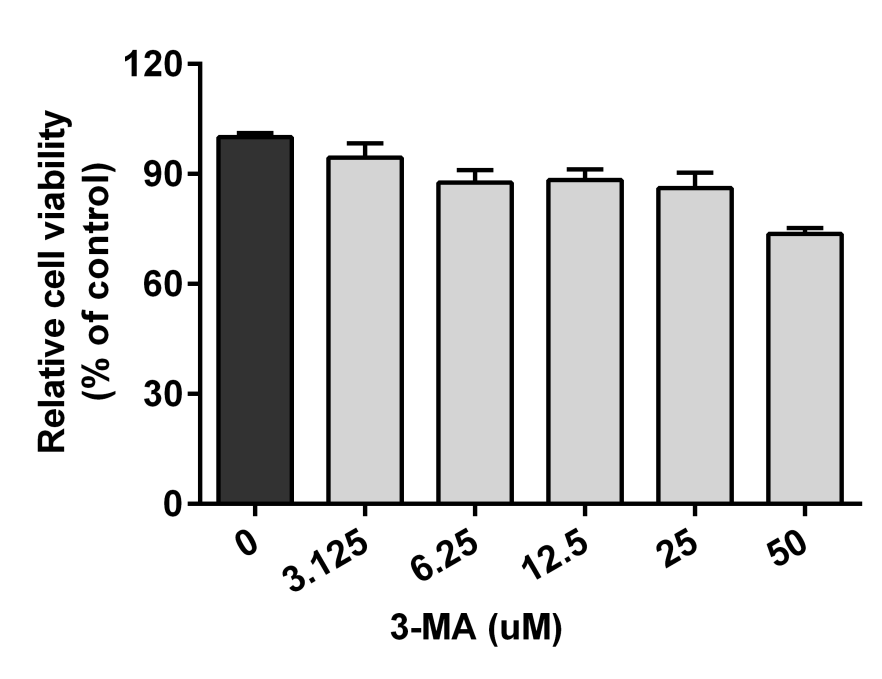


Figure S1

a. Cell viability was determined with the CCK-8 assay under different concentrations of 3-MA.


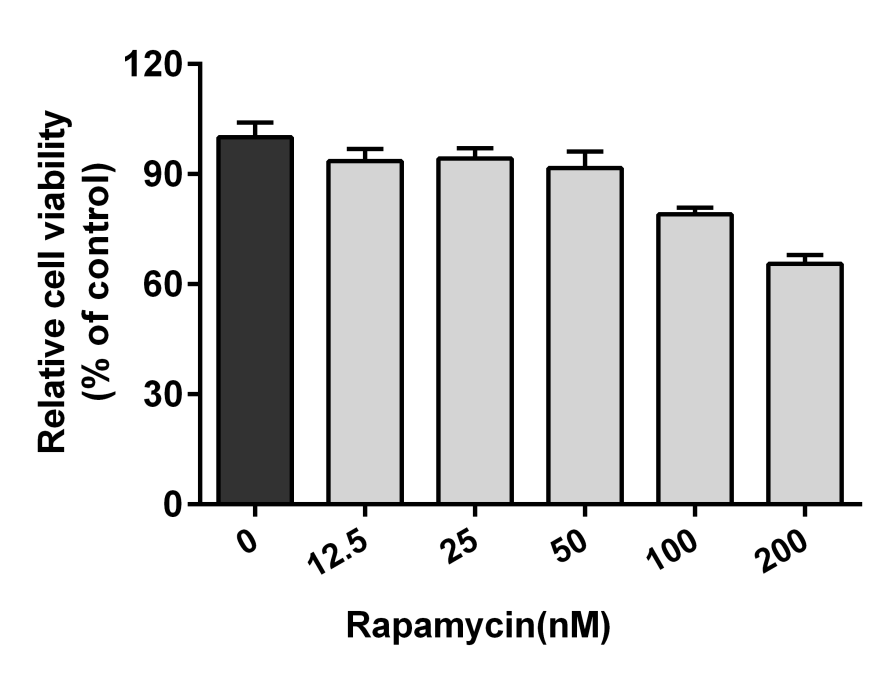


Figure S2

a. Cell viability was determined with the CCK-8 assay under different concentrations of Rapamycin.
